# Cancer, obesity, and legitimation of suggested lifestyles: a libertarian paternalism approach

**DOI:** 10.3332/ecancer.2015.588

**Published:** 2015-10-29

**Authors:** Giovanni Boniolo, Vincenzo Rebba

**Affiliations:** 1 Institute for Advanced Science, Technische Universität München, Lichtenbergstr. 2a, Garching D-85748, Germany; 2 Dipartimento di Scienze Biomediche e Chirurgico Specialistiche, University of Ferrara, Via Fossato di Mortara, 64A, Ferrara 44121, Italy; 3 Department of Economics and Management, University of Padua, via del Santo 33, Padua 35123, Italy

**Keywords:** cancer, citizen autonomy, deliberation, health care costs, libertarian paternalism, obesity, policies

## Abstract

We know that around 30% of all cancers are preventable. We also know that there is clear evidence of the causal relations between obesity and cancer. This means that there could be lifestyles that could prevent obesity and, thus, cancer. Yet, who legitimises these lifestyles and on which ground? Should citizens be free to accept or not to accept policies concerning them? This is a problem faced within what has been named libertarian paternalism. We discuss it, also proposing a version that we call deliberative libertarian paternalism, showing how important this problem is for a proper framing of the lifestyle policies concerning obesity and, thus, cancer prevention.

## 1. Introduction

It seems clear that around 30% of all cancers are preventable (See www.cancerresearchuk.org/health-professional/cancer-statistics/risk/preventable-cancers). In particular, they are preventable acting on their causes. And we also know that obesity and lack of physical activity are risk factors [1–5]. This means that we should suggest lifestyles indicated to avoid obesity and to increase physical activity. But why and on which basis should a citizen, endowed with free will, accept those suggestions? This is not a trivial problem, especially now that a citizen, be it an actual or potential patient, may acquire the awareness of his/her autonomy in his/her relationship with the clinician. There is an extremely lively debate on this issue since it is not so easy to reconcile institutional or even state suggestions concerning healthy lifestyles and the liberty of the individual to live in the way that he/she thinks is best suited to his/her beliefs and perception of life.

Here, we will discuss this topic, focusing on obesity prevention, and thus on cancer prevention, and on the tension between institutional or state policy and citizen autonomy. This means discussion about what is called libertarian paternalism, as we will show.

Regarding obesity, we know that it is a global health problem, affecting people of all ages. The World Health Organisation (WHO) estimates that there were more than 1.4 billion overweight adults and at least 500 million obese adults worldwide in 2008 [6]. Excess body mass (obesity and overweight) is a known risk factor for a range of diseases including type 2 diabetes, cardiovascular disease, hypertension, and several cancers (colorectum, pancreas, gallbladder, oesophagus adenocarcinoma, kidney, endometrium, and postmenopausal breast cancer) [7–9]. Obesity is caused by high-fat diets, sedentary living, genetic factors, and disorders of the endocrine system [10]. The economic impact of obesity, in terms of both direct costs due to health care and indirect costs related to lower productivity and wages, has also been estimated to be very large. Individuals who are obese incur health care expenditures at least 25% higher than those of normal weight persons and obesity is estimated to be responsible for 1–3% of total health expenditure in most countries (5–10% in the United States) [11] (By one recent estimate, the U.S. spent in 2005 $190.2 billion on obesity-related health care expenses, that is, around 20.6% of national health expenditures (both private and public), an amount that is considerable higher than previous estimates [19].). Furthermore, the combined medical costs associated with treatment of obesity-related preventable diseases are projected to increase by $48–66 billion/year in the United States and by £1.9–2 billion/year in the United Kingdom by 2030 [12]. As a result, a number of contributors to the scientific literature and policy-makers have been proposing eligibility criteria and funding methods in health care predicated on individual responsibility. To date this approach has had a negligible impact on public health systems: the choice adopted is to avoid discrimination in access to health services, while calling for public interventions intended to influence behaviour [13] (Some U.S. states have introduced charges and copayments that can be waived when beneficiaries comply with specific wellness targets. The most prominent example of such charges is the ‘fat tax’ introduced in Alabama where, since 2009 state employees must receive medical screenings (including a calculation of their BMI) for several conditions. Those who are considered obese or who exhibit other negative health factors will have a year to get in shape. If they fail to do so, they will have to pay $25/month more for their health insurance [18].). Governments and public health agencies have increasingly enacted policies meant to prevent obesity and promote healthy lifestyles [14, 15]. Surveys of national policies covering OECD and EU countries show that governments are stepping-up efforts to promote a culture of healthy eating and active living [11, 16, 17]. Governments are generally reluctant to use regulation and fiscal levers in this field because of the complex regulatory process, the enforcement costs, and the likelihood of confrontation with key industries. In particular, taxes on calories affect consumers indiscriminately, are difficult to implement (measuring the tax base in terms of fat or sugar content is not always practical), and may have regressive effects (deterring low-income families from getting enough nutrition), so they can have high political and welfare costs [18]. Most governments have adopted initiatives aimed at school-age children, such as changes in school meals and vending machines, better facilities for physical activity, and health education. Many also disseminate nutrition guidelines and health promotion messages such as encouraging active transport – cycling and walking – and active leisure. New legislation on food labelling and tighter regulation of food advertising has been recently implemented in a number of OECD countries. Moreover, several OECD countries have increased their use of financial incentives linked with health and wellness objectives and a number of countries are increasingly relying on multi-stakeholder platforms and cooperation with private sector (in particular, food and beverage industry) in their efforts to counter obesity [20] (In the United Kingdom, for instance, a Public Health Responsibility Deal was launched in 2011, in which the government sets targets and priorities and business partners make voluntary pledges contributing to the strategy. This evidence has polarised views on the role of private sector involvement in public health policy, but evaluations are only beginning to emerge.).

However, the most utilised policies designed for the population level (health promotion, information campaigns, and financial incentives) have often proven ineffective over the long term, while tailored treatment programmes that factor in stressors and temptations of the real world, using insights from behavioural research, are showing some success [21, 22]. Several of these programmes are based on a relatively new approach labelled libertarian paternalism (hereafter, LP) which aims at ‘nudging’ individuals into acting in their (and society’s) best interest, pursuing an acceptable compromise between the corrective objective (promoting healthy lifestyles) and respect for the freedom of individual choice [23, 24]. According to this approach, it is possible to reconcile respect for individuals’ autonomy with paternalistic interventions aimed at making individuals behave in health-promoting (and, more generally, welfare-promoting) ways. This can be done by designing choice situations in ways that – given individuals’ psychological tendencies, limitations and biases – are apt to generate health-promoting choices. Individuals retain the freedom to choose unhealthy (or self-harming) options, thereby making this kind of paternalism liberty-preserving. This suggestion has prompted a long-standing debate, partly reviving the old debate about the legitimacy of paternalism in public policy.

In the following sections, we first present a short description of LP and draw a link between LP, obesity and cancer prevention. In Section 3, we discuss some of LP’s shortcomings. Hence, in Section 4, we argue that those charges are not fatal to the theory, but that they still point to issues which deserve serious consideration. For these issues, we eventually suggest a ‘deliberative’ solution; then, we provide some examples of deliberatively justified LP policies to tackle the specific issue of obesity, contributing to reduce the cancer risk. Section 5 lists our conclusions.

## 2. Libertarian paternalism for obesity and cancer prevention

### 2.1 What is libertarian paternalism?

People make inferior choices. Choices ‘they would change if they had complete information, unlimited cognitive abilities, and no lack of willpower’ [25]. Furthermore, most of the times, structural features of the situation in which individuals take their decisions influence those decisions. These structural features can be effectively changed in ways that increase decision-makers’ welfare (health being a component of welfare) ‘as judged by themselves’ [23], [25]. In Nudge (2008), Thaler and Sunstein’s best seller, the authors argue that whenever the changes that are introduced do not significantly alter incentives or rule out some option altogether, then the kind of paternalism that is implied by the introduction of the new situation is genuinely libertarian, that is, unproblematically uncoercive. To clarify this point, consider the following example. Suppose the director of food services for a large city school system realises that the way in which food or dinnerware are arranged significantly influences the choices school kids make. By selecting a particular arrangement over another, he/she can affect the way kids choose the food they are going to eat. If he/she selects the arrangement properly, he/she can affect children’s choices in a way that is conducive to healthier eating behaviours and healthier diets (Rozin *et al* (2011) [27] find that people might eat 8% to 16% less if food is made only slightly more difficult to reach (for example reducing its proximity by 10 inches), while Wansink and van Ittersum (2013) [28] show that the size of plates and bowls strongly influences how much one eats. Therefore, our director may consider both to putting healthy food before less healthy meals and providing larger dinnerware near the more healthy food items, while providing smaller dinnerware near the less healthy items.), which will in turn result in better health and prospectively in reduced societal costs (Kantor *et al* (2015) [29] show that being obese or very overweight during teenage may double the risk of developing colorectal cancer by the time one is middle-aged. This highlights the importance of nudging adolescents toward healthy diets.). Anyone who has responsibility for organising the context in which people make decisions is, in Sunstein and Thaler’s words, a choice architect.

More generally, choice architects can design choice situations with the aim to increase the number of decisions that promote (or are thought to promote) the health and welfare of the choice makers, respecting their freedom of choice. In the scenario just described, children can still opt for choices that make them worse off, and therefore, their liberty is unencumbered by a choice architecture acting upon paternalistic grounds. A policy, thus, counts as libertarian paternalistic if, while refraining from reducing the array of choices people have, it is selected with the goal of influencing the choices of affected parties in a way that will make those parties better off, as judged by themselves: the intrusion is not to be considered coercive as long as it does not preclude some options altogether.

Any situation in which some kind of cognitive bias can operate is a good candidate for the deployment of LP measures. These biases impair people’s decision-making in a number of ways and in a series of actual situations. Building upon behavioural insights, the intuition underpinning LP is that people would not consent to choices and behaviours that they end up enacting due to the significant ways in which they cognitively lack, and hence, adjustments meant to counteract those biases are not only unrestrictive of individual liberty but they also embody one’s own set of preferences better than one’s own behaviour does. Such adjustments constitute the toolbox of LP policy-making. According to Sunstein and Thaler (2003) people’s choices will be affected by ‘default rules, framing effects and starting points’. Essentially, all of these are systematic ways in which we do not live up to our own behavioural standards. Everyone is more or less lazy, and, LP has shown, making the healthiest (or most welfare-enhancing) choice the easiest to pick will ‘nudge’ people into taking it.

Given that obesity is associated with increased risks of cancer and has high individual as well as societal costs, relying on LP-like policies could benefit both individuals and society while at the same time preserving everyone liberty to indulge in self-harming behaviours.

### 2.2 Some examples

A host of policies for cancer prevention have been proposed that allegedly fall under the heading of LP. These measures promote evidence-based interventions (e.g. breast, cervical, and colorectal cancer screening) and include the use of incentives, introduction of default options (an opt-out enrolment rule), appropriate feedback throughout the decision-making and behaviour completion process, and clear presentation of complex choices [26] (These LP measures are often tailored according to targeted populations in order to nudging underserved – typically low income and minority – populations.).

Here, we focus on LP policies meant to prevent obesity, thereby contributing to reduce the cancer risk (Most data about whether losing weight or avoiding weight gain prevents cancer come mainly from cohort and case–control studies. Even though data from these observational studies can be difficult to interpret, owing to individual heterogeneity (e.g. it is often difficult to evaluate whether the weight loss was intentional or related to underlying health problems), some studies have found decreased risks of breast cancer and colon cancer among people who have lost weight [35].). We believe that only a few of them would qualify as genuine LP policies, either because they are often, at a closer inspection, information campaigns by a different name, or because they entail the introduction of non-negligible economic incentives (In this respect, we do not consider as LP policies some of the interventions considered by Dubé (2010) [36].). We consider here some examples of these LP policies, mostly focused on behavioural change.

**Shopping trolley re-design.** In England, 65% of adults eat less fruit and vegetables than they ought to [30], especially if they should eat them in order to tackle obesity issues. Concerning this situation, the Cabinet Office’s Behavioural Insights Team (so-called Nudge Unit) suggested following the lead of the New Mexico State University College [31] (The evidence cited concerning the experiment conducted by Collin Payne at the New Mexico State University College of Business is said to be contained in personal communication between Payne and the Behavioural Insights Team.). Payne found that putting a line of yellow tape with a sign designating the part of the trolley to be devoted to fruits and vegetables increased the amount of fruits and vegetables purchased without affecting the overall profit for the retailer. It is unclear how far a policy inspired by such evidence would qualify as LP. In fact, the only ground for the claim that people are being made better off, as judged by themselves, by such a policy, lies in the assumption that people know they should be eating five portions of fruits and vegetables a day (This, of course, unless one assumes that everyone prefers being healthy over eating junk food.). Nonetheless, the policy would indeed be noncoercive and it would entail no change in the architecture of economic incentives. (An example of this policy is the collaboration between Asda supermarkets and the Department of Health’s Change4 Life campaign, whereby social norm messages were advertised on trolleys [31]. In 2012, the British government launched a ‘calorie reduction pledge’ within the Public Health Responsibility Deal providing a mechanism for the food and drink industry to make and record its contribution to helping the population to adopt healthy diets. The responsibility deal in the United Kingdom involves also other private–public partnerships on promoting physical activity, health at workplace and reduction in salt and alcohol intake (https://responsibilitydeal.dh.gov.uk/). Private-public partnerships on reducing childhood obesity are gaining ground also in the United States, as reported by Sunstein (2013) [37].)

**Wellness programmes at the workplace.** A large, and rapidly increasing, number of employers worldwide are providing incentives for participation in wellness programmes (currently 62% in the United States and 16–41% elsewhere) [32, 33], and, as we know, physical activity prevents and counterbalances obesity. Within this framework, a LP policy which has been proposed for UK by Julian Le Grand – former Downing Street adviser – was to require employers over a certain size to offer their employees exercise facilities and a time during the working week to exercise, but which employees could opt out of if they so wished [34]. Making opting in the default is clearly a mechanism meant to defy laziness. However, in this case, though no actual monetary incentives are involved, (i) the requirement that employers offer time off duty to their employees is coercive towards the employer and (ii) employees are non-negligibly incentivised to exercise for the mere reason that doing so will entail paid hours off work (technically, the opportunity cost of the choice, that is, forgoing hours of work, while still being paid, is negative, making the choice a no-brainer).

**Nudging physical activity.** Despite the long-term benefits of exercise, a key problem is the difficulty of translating current motivation into long-term behavioural change. While for many people the prospect of exercising for three months and improving their health may seem attractive today, the daily decision to actually do the exercise during that period is a much more difficult one. In order to investigate effective ways of overcoming this lack of commitment, Goldhaber-Fiebert *et al* (2010) conducted a randomised controlled experiment among 619 individuals living in the United States aged 18–69 years who were randomly shown a default contract duration (8 weeks, 12 weeks or 16 weeks) [38]. They found that those shown longer default duration were more likely to choose a contract of a longer duration. Furthermore, individuals who were nudged into longer contracts did not commit to less exercise per week, nor did they choose smaller financial penalties for not fulfilling their longer contracts. These findings demonstrate that nudges are effective in changing the length of the exercise commitment contracts users choose without altering their other contract features (e.g. frequency of weekly exercise, size of financial stakes, etc.). Though this looks very much like a case of genuine LP, introducing the aforementioned architecture of choice would have made sense to the owners or the managers of the gyms irrespective of the actual health benefit that is enjoyed by the customers, which makes the introduction, via policy-making, of a regulation to display longer contract durations pointless.

## 3. Shortcomings of LP

### 3.1 Externalities, internalities, and self-control problems

In standard politico-economic terms, whenever individual behaviours have costs that ‘spill over’ onto third parties, that is, they produce externalities, the government has some entitlement to intervene on the grounds of the so-called ‘harm principle’. As famously articulated by John Stuart Mill, this principle holds it that:

[T]he only purpose for which power can be rightfully exercised over any member of a civilized community, against his will, is to prevent harm to others. [58]

When individuals bear all costs and benefits of their own actions, instead, there is little ground for government intervention, even in cases where self-inflicted harm is at stake. Thus, in case, obesity was shown to produce substantial externalities, the ‘harm principle’ would apply and allow for coercive forms of paternalism.

Fiscal or subsidy externalities are the most important externalities linked to obesity: when health care is funded through public expenditure (as in most European countries), the additional health care cost needed by an obese person is borne by taxpayers; if an insurance plan is involved (as in the United States), the cost will be shared among all those covered by the plan, who pay a premium for their care [11]. However, the obesity externality, although more important in Europe than in the United States, seems rather modest and is therefore unlikely to be a good reason for the implementation of LP policies [39, 15, 40].

New paternalists, however, argue that there is a special kind of ‘within-person externalities’ – which they call internalities – at stake when people engage in self-harming behaviour of specific kinds (The term ‘internality’ was introduced by Herrnstein *et al* (1993) [41].). An internality occurs when a person underestimates or ignores a consequence of her own behaviour for herself. Relying on a dubious language of ‘multiple selves’, LP contends that incontinent present selves might be construed as imposing costs on future selves. This could be regarded then as grounding the same kind of governmental interventions that are seemingly non-controversial in the presence of externalities (Adopting this particular perspective, some economists [42] have developed a theory of ‘sin taxes’, that is, taxes which counteract over-consumption by consumers with self-control problems while at the same time redistribute income to consumers with no self-control problems.). The internality issue is strictly linked to ‘self-control’ problems typically characterising food consumption. Individuals are usually informed about the dire consequences of overweight and obesity on health (For example, according to the 2005 Eurobarometer Survey, less than 9% of European adults declare not to find it easy to follow a healthy diet due to information problems [15].). Nevertheless, they seem to ignore sound advice on health and nutrition: even if they understand the long-term negative consequences of eating too much or not exercising, this counts less than the immediate gratification they obtain from consumption [43]. This type of time-inconsistent behaviour (hyperbolic discounting) – with people constantly re-optimising over the short term, and quickly abandoning the long-term plan that was originally optimal – is affected by the individual’s psychological state at the moment the preference is expressed and it has been linked to the problems of addiction (For a survey on hyperbolic discounting, see Frederick *et al* (2002) [47].). This inconsistency of preferences over time is furthermore what makes people with poor self-control particularly vulnerable to the influence of an obesogenic environment [11] (As Cutler *et al* (2003) argue, technological innovation that reduces the time costs of food preparation affects food consumption by reducing the price of food and by reducing the delay before consumption [48]. While the price reduction affects all, the reduced time delay will mostly affect individuals with self-control problems, who will spend more than is optimal on food and incur a welfare loss if the health costs of additional weight due to overconsumption are greater than the welfare gain from lower costs of time food preparation.). LP interventions on consumers’ selection of food items should exploit both present-biased preferences and status quo bias, introducing a negligible immediate cost to selecting unhealthy meals (e.g. in the cafeteria example, the dessert section, unlike the fruit section, may require queuing a second time) (Down *et al* (2009) analyse the effect of a LP experiment on consumption of fast food low and high-calorie sandwiches [49].). If equating standard externalities to internalities makes sense, then LP arguments have a bite and LP-based health policies ought to be designed and implemented. The analogy is however harder to draw than it seems at a first sight.

As pointed out by Whitman (2006) and Sugden (2008), internalities are about avoiding the more serious harm between the one currently inflicted to future selves and the one that would be inflicted to present selves in case paternalistic policies (of any sort) would be put in place [44, 45]. The assessment of the harm one wants to avoid is articulated in terms of the comparative inefficiency in the allocation of resources in the two scenarios. In Sugden’s words (2008):

[…] before we can conclude that a paternalistic third party could do better, we need to know (or rather, the third party needs to know) which preferences reflect well-being and which do not. Is the person’s well-being greater if he saves or if he spends? If he insures or if he doesn’t? If he diets or if he indulges? […]

Granting this point, then it can be the case that ‘some, though probably not all, of the present self’s future costs will already have been internalised through intrapersonal bargains’ [44]. If internalities coming from present selves’ behaviours are already accounted for due to intrapersonal bargains, there is little ground to nudge people into making decisions that go to the exclusive advantage of their future selves. In a sense, this would result in individuals’ underconsumption of goods they value, which will in turn mean overall diminished welfare.

As argued in Schiavone *et al* (2014), we believe that the issue raised by Whitman and Sugden is a serious one [45]. This issue underscores the main problem of LP, namely that it fails to provide conclusive reasons why some specific set of individual preferences (namely future-selves’ preferences) is to be considered preferable to others.

Next, we briefly address a second issue potentially hindering the tenability of LP as a framework for the development for healthcare policies in general and obesity-related policy more specifically.

### 3.2 Policymakers’ inadequacy

Let us define meta-choices as choices over architectures of choices. The very possibility of having someone decide on what architecture of choices will be available (and to some extent on what choices will actually be taken) opens up a whole array of issues related to the selection of those who operate meta-choices. First, given the assumption underlying LP that everyone ought to take decisions in the epistemically best position possible, it seems only fair that society demands the same epistemic standards for decision-makers who are in charge of meta-choices. In particular, since individual decision-makers are nudged into deciding as ‘if they had complete information, unlimited cognitive abilities, and no lack of willpower [23] choice architects would, *a fortiori*, be required to be both informationally, cognitively and motivationally unbiased.

Addressing this worry, Sunstein and Thaler dismiss it relatively quickly claiming that ‘sunlight is the best of disinfectants’ [25], and therefore, transparency and accountability will provide choice-architects with the right kind of incentive to rely on the best information available, to put their best cognitive efforts into making the decision and not to be captured by individual or private group interests.

However, the stakes for obesity-related policymaking just seem too high to simply ask for transparency and accountability without any proper procedural account of how choice-architects ought to be chosen and why they would then be legitimately taking their meta-choices.

In Section 4, we propose how this could be done in a way that, we contend, manages to both weaken the objection articulated in paragraph 3.1 and effectively address the concern that nudgers might hijack meta-choices just highlighted.

## 4. Deliberative libertarian paternalism in health care policies

### 4.1 Epistemic and pragmatic superiority of deliberative libertarian paternalism

We have rehearsed some of the reasons why the most urgent problem affecting LP is its incapability to articulate the rationale for choosing one intrapersonal set of preferences (that belonging to future selves) over some other. In Schiavone *et al* (2014), we proposed a deliberative solution [46] (Let us remind that deliberation is the procedure through which people, starting from different initial positions, try and reach a shared decision via a debate based on rational arguments and counter-arguments. For a discussion on deliberative democracy and its story, see Boniolo (2012) [50].) to this troubling issue, providing epistemic and pragmatic reasons why what we call ‘deliberative libertarian paternalism’ (DLP) is superior to LP (A similar solution has been put forward by Anand and Gray (2009) [51].). Let us summarise our argument.

First, a so-called ‘ideal conversation’, that is, a debate in which everyone is only constrained by the strength of other people’s arguments, can expose internal inconsistencies in individuals’ positions. This would in turn help constructing tenable (meaning reasonable) personal preferences over meta-choices. As Boniolo put it:

A well-conducted deliberative process is never unsuccessful, because the rational discussion by itself leads participants to reflect upon the tenability of their own and their opponents’ positions. In short, there is always a deliberative result beyond any desired consensus. [50].

Secondly, the application of deliberative democratic standards to the selection of LP-inspired obesity-related policies would defeat the issue of ‘bad’ nudgers highlighted in Section 3.2. As it stands, LP is a policy-making device that groups of politically appointed experts rely on. However, this kind of technocratic (or ‘expertocratic’) solution provides no reason or explicit set of criteria for the selection of the experts that end up being selected. Furthermore, such a procedural arrangement is clearly inconsistent with the ideal of equality in freedom, a good common to most, if not all, democratic politico-theoretical accounts [52].

We propose instead to directly engage lay citizens in obesity-related policy-making. There are risks as well as benefits linked to our proposal. The easiest risk to identify is the standard elitist objection to direct participation to self-government initiatives: people’s preferences concerning architectures of choices would be uninformed. However, extensive empirical research has shown that through an appropriate process of tutoring of people’s preferences, deliberation could both build upon the best knowledge available and be sensitive to people’s actual preferences. Giving people direct access to healthcare policy-making, obesity being a specific case here, any decision will simply not assume anything about people’s own conception of welfare, thus turning the ‘as judged by themselves’ clause put forth by LP into a reference to the actual judgment of a properly sampled group of stakeholders rather than to the counterfactual reconstruction of every individual set of preferences.

To sum up, deliberative tools applied in this context might help developing choice architectures that comply better with people’s tutored preferences and which are more politically legitimate as well.

### 4.2 Deliberatively justified LP policies to control obesity

Deliberation could play a significant role in implementing LP policies aimed at obesity prevention such as those mentioned earlier. In those instances, the ideal of deliberation would demand that relevant stakeholders convene and be given certified and understandable information concerning obesity, overweight, their negative clinical and oncological consequences, their economic and social costs, and the effectiveness of likely strategies for the intervention. We envisage, following this first phase, presenting participants known arguments in favour and against the application of specific policies.

We identify one main source of potential problematic issues in the application of our proposal. Engagement initiatives ought to be as inclusive of different social and cultural backgrounds as possible, and this might reveal difficult due to the technical knowledge at stake during the tutoring phase. To tackle this issue, we propose devising the deliberation so as to have informational material that are both in plain language and available for revision on account of participants doubts and objections. Furthermore, we propose using facilitation techniques to enable the participation of subsets of the population unlikely to be familiar with the argumentative style that is proper of ideal conversations.

## 5. Conclusion

In recent years, since the seminal contribution of Thaler and Sunstein (2003) proposed a new approach to public policy based on behavioural economics, the idea of LP has taken hold, not only in academia but also in government and business (especially in the US and UK). LP is appealing since it proposes a set of seemingly simple, low-cost solutions that do not require regulation and can be applied to a wide array of behavioural problems, including unhealthy diets and sedentary habits, even though concerns have been raised about the effectiveness and coherence of the approach [32, 53–57].

These concerns notwithstanding, LP suffers from at least two theoretical issues that we rehearsed here: (i) long-term selves’ preferences are systematically chosen vis-à-vis inconsistent short-term selves’ preferences and no compelling rationale is presented for this choice; (ii) the selection of choice-architects Sunstein and Thaler propose fails to tackle effectively concerns for political legitimacy and competence. We therefore propose to rely on a deliberative version of LP and argued that applying DLP to obesity-related policy-making would address both concerns more than satisfactorily.
